# S3. CHILDHOOD TRAUMA AND COGNITIVE FUNCTIONING IN SCHIZOPHRENIA SPECTRUM DISORDERS: EFFECT OF FREQUENCY AND TYPE OF CHILDHOOD TRAUMA

**DOI:** 10.1093/schbul/sby018.790

**Published:** 2018-04-01

**Authors:** Nina Morkved, Erik Johnsen, Rune Kroken, Rolf Gjestad, Dagfinn Winje, Jens Thimm, Farivar Fathian, Else-Marie Løberg

**Affiliations:** 1 Mosjoen District Psychiatric Centre, Helgeland Hospital; 2 Haukeland University Hospital, University of Bergen; 3 Haukeland University Hospital; 4 University of Bergen; 5 University of Tromsø; 6 NKS Olaviken Gerontopsychiatric Hospital

## Abstract

**Background:**

Cognitive impairment is a core feature of schizophrenia spectrum disorders (SSDs). Exposure to childhood trauma (CT), defined as physical, sexual and emotional abuse, and physical and emotional neglect, has been associated with SSDs across study designs and populations. Possibly, there is a relationship between exposure to CT and cognitive impairment in individuals with SSDs. Research has shown that a history of CT may be related to decline in cognitive performance in the general population, as well as in SSDs, whereas other studies have failed to find evidence for an association between CT and cognitive impairments in patients with SSDs. Findings on the relation between CT and cognitive impairment in individuals with SSDs is not conclusive, and a minority of the studies to date have examined the effects of frequency and severity of CT subtypes in SSDs and the relation to cognitive abilities. We hypothesize that there will be a negative relationship between the frequency and severity of CT and cognitive functioning, possibly in a dose dependent matter. CT subtypes may influence this relationship.

**Methods:**

The present study is part of the Bergen Psychosis project 2 (BP2), Haukeland University Hospital, Norway. Patients were recruited at the Medical University in Innsbruck, Innsbruck, Austria; Stavanger University Hospital, Stavanger, Norway; and Haukeland University Hospital, Bergen, Norway, and gave informed consent to participate. To be included, patients had to meet ICD-10 criteria for SSDs (F20-F29), be > 16 years of age, and score ≥ 4 on at least one of the psychosis items on the Positive and Negative Syndrome Scale (PANSS). Childhood trauma (physical, emotional, sexual abuse, and physical, emotional neglect) was measured by the Childhood Trauma Questionnaire Short-Form (CTQ-SF). Cognitive functioning was examined by means of a comprehensive neuropsychological test battery. The following cognitive domains were assessed: verbal and visuospatial abilities, learning, memory, attention and working memory, executive functioning, and processing speed. The assessments were completed within three months of inclusion to the study.

**Results:**

The relationship between the frequency and severity of CT and cognition will be examined, in addition to the possible influence of CT subtypes. Preliminary findings will be reported.

**Discussion:**

The clinical implications of our findings will be discussed.

